# IPEX Syndrome with Normal FOXP3 Protein Expression in Treg Cells in an Infant Presenting with Intractable Diarrhea as a Single Symptom

**DOI:** 10.1155/2020/9860863

**Published:** 2020-09-09

**Authors:** Ali Al Maawali, Beata Derfalvi, Johan Van Limbergen, Andrew Issekutz, Thomas Issekutz, Hasan Ghandourah, Mohsin Rashid

**Affiliations:** ^1^Department of Paediatrics, Faculty of Medicine, Dalhousie University, IWK Health Centre, Halifax, Nova Scotia, Canada; ^2^Division of Immunology, Department of Paediatrics, Faculty of Medicine, Dalhousie University, IWK Health Centre, Halifax, Nova Scotia, Canada; ^3^Division of Pediatric Gastroenterology and Nutrition, Emma Children's Hospital, Amsterdam University Medical Centers, Amsterdam, Netherlands

## Abstract

IPEX (immune dysregulation-polyendocrinopathy-enteropathy-X-linked) syndrome is a rare, potentially fatal multisystem disorder caused by mutations in the *FOXP3* gene. This can lead to quantitative or functional deficiency of regulatory T cells (Treg), thereby affecting their immune-suppressive actions which can in turn cause autoimmune and inflammatory disorders. We describe an infant with IPEX syndrome with unremarkable maternal family history whose only presentations were severe diarrhea and malnutrition. The patient had a normal percentage of Treg cells and FOXP3 protein expression, but further testing revealed a hemizygous missense mutation in the *FOXP3* gene. IPEX syndrome should be considered in young children even if severe intractable diarrhea is the only symptom with no other autoimmune manifestations. Sequencing of the *FOXP3* gene should always be considered for accurate diagnosis to look for mutations even in the face of normal FOXP3 protein expression in the Treg cell.

## 1. Introduction

IPEX (immune dysregulation-polyendocrinopathy-enteropathy-X-linked) syndrome is a rare multisystem disorder that often presents in early childhood and can be fatal. It was first described by Powell et al. in 1982 [1]. Immune dysregulation is the hallmark of this disorder. Human immunological self-tolerance on the periphery is controlled by a subset of T lymphocyte cells called the regulatory (Treg) cells [2]. They play a pivotal role in suppressing autoimmunity and atopy by suppressing self-reactive lymphocytes and preventing excessive reactions to self- and foreign antigens [2, 3]. The Treg cell development and function are dependent on the expression of a protein called forkhead box protein 3 (FOXP3). *FOXP3* is a transcription factor located on chromosome Xp11.23 and is considered a master switch gene for these Treg cells. The absence or dysfunction of *FOXP3* can cause qualitative or functional deficiency of Treg cells, thus leading to immune dysregulation and IPEX syndrome [2–5]. IPEX syndrome can be misdiagnosed and hence may be underreported [6].

## 2. Case Presentation

A 5-month-old male was admitted with severe diarrhea and failure to thrive. The baby was born at term after an uneventful pregnancy and delivery with a birth weight of 2.97 kg. He was exclusively breast-fed. At 2-1/2 months of age, he developed frequent, large volume, watery stools with occasional blood streaks. There was no fever, emesis, or any infectious contacts. The family history was only significant for two paternal second-degree relatives with celiac disease and one-second degree relative with a thyroid disorder and type 1 diabetes mellitus.

On initial examination, the child appeared ill, pale, malnourished, and dehydrated. The weight was 5.19 kg (<3rd percentile), length was 59.7 cm (<3rd percentile), and head circumference was 40.5 cm (between 3rd and 10th percentile). Other than some diaper rashes, the physical examination was unremarkable with no atopic dermatitis or icthyosis.

The results of initial investigations are shown in Table 1. The absolute eosinophil count was only slightly elevated. Immunoglobulins including IgE were normal. The serum albumin was low, consistent with the protein losing enteropathy. Stool examination did not reveal any pathogens. Stool electrolytes showed secretory diarrhea. The antinuclear antibody and direct antiglobulin were negative.

Endoscopy and biopsies revealed acute and chronic inflammation, marked autoimmune enteropathy with total villous atrophy, and abundant CD3^+^ T- and CD20^+^ B-cell aggregates in the small intestine and colon.

**Figure 1:**
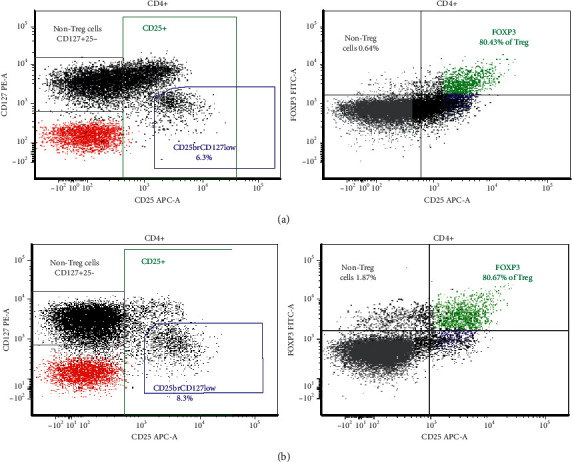
FOXP3 expression in Treg cells measured by flow cytometry: (a) control; (b) patient.

Further immunological workup showed intact IL-10 receptor function since endotoxin-induced tumor necrosis factor (TNF) production in mononuclear cells was suppressed by IL-10. Initial T- and B-cell phenotype analysis was normal. The percentage of circulating Treg cells and FOXP3 protein expression in these cells measured by flow cytometry were also normal (Figures 1(a) and 1(b)).

Analysis of all coding regions and exon/intron boundaries of the *FOXP3* gene was carried out. This revealed a hemizygous missense mutation (c.1190G > A, p.Arg397Gln) which would result in a nonfunctional FOXP3 protein and Treg cells, similarly in the patient's mother, who is a healthy career. The anti-islet cell antibody was negative, insulin-like growth factor-1 level was normal, and the patient never developed type 1 diabetes mellitus. Thyroid function was also normal.

The patient required intensive resuscitation with intravenous fluids. He was then managed with parenteral nutrition and high-dose intravenous corticosteroids. He was maintained on oral prednisone and tacrolimus. The clinical condition gradually improved, and he was able to initiate oral intake with an elemental formula in a few weeks.

The patient had a matched unrelated hematopoietic stem cell transplant (HSCT) with reduced intensity conditioning including alemtuzumab, fludarabine, and melphalan at 8 months of age. Early transplant period was complicated with donor Epstein–Barr virus (EBV) reactivation treated with rituximab. He developed autoimmune hemolytic anemia. Initially, enteropathy significantly improved; however, colitis recurred 3 months after the transplant when, unfortunately, there was decreasing mixed chimerism and secondary bone marrow rejection. Donor lymphocyte infusions were unsuccessful, and the patient required a second transplant at 14 months of age from the same donor with busulfan, fludarabine, and alemtuzumab conditioning this time.

Two years after the second HSCT, the patient has mixed chimerism and 65% of donor haematopoiesis but 91% of donor Treg cells (Table 2). The gut function gradually recovered, and graft-versus-host disease was ruled out on repeated gut biopsies. Currently, he is doing well, except having eczema of typical distribution and morphology seen in toddlers and responding to high-potency topical corticosteroids. He is immunocompetent and has sustained protective antibody titres to several vaccine antigens.

## 3. Discussion

IPEX syndrome is a rare but potentially fatal disorder requiring a high index of suspicion for a timely diagnosis. The commonest mode of presentation is with intractable diarrhea, growth problems, dermatitis, and autoimmune endocrinopathy [6, 7]. Histological finding in the IPEX syndrome, as with most other autoimmune enteropathies, is variable with the commonest finding being a graft-versus-host disease-like pattern associated with positive antienterocyte antibodies [8]. Screening the enteropathy-related autoantibodies (antivillin, antiharmonin, and antienterocyte antibodies) which are 8–92% positive in sera of IPEX patients could have helped the diagnosis in our patient as well [9, 10]. Additional clinical features of autoimmunity such as Coombs-positive hemolytic anemia, thrombocytopenia or neutropenia, hypothyroidism, and interstitial nephritis [6] were tested; however, our patient is unique since his only presentation was severe diarrhea. There is a wide spectrum of disorders that can present with severe life-threatening diarrhea in infants, and these should be considered in the differential diagnosis [11].

The initial laboratory findings may show eosinophilia, neutropenia, anemia, or thrombocytopenia as a result of autoimmune phenomena [12], and other autoimmune abnormalities should be sought, including anti-islet antibodies and antithyroid antibodies. Our patient had no autoimmune phenomenon apart from enteritis [7, 8] and had normal serum IgG level despite severe protein loss through the gut. Serum IgE and IgA may be elevated in IPEX syndrome, but our patient had normal levels [6, 8, 13]. Lymphocyte subsets are typically not diagnostic as B- and T-cell subsets are normal in most patients with IPEX syndrome. The Treg cell immunophenotyping quantitative analysis via flow cytometry, when compared with an age-matching control, may show absence of Treg cells; however, the presence of the Treg cell and *FOXP3* expression may not exclude IPEX syndrome, as demonstrated in our patient [4, 14]. Immature Treg cells can still be generated in the presence of a mutation and alteration in the *FOXP3* gene, and intact *FOXP3* is essential for the development and, more importantly, the function of Treg cells [13]. Gene sequencing to identify a mutation in *FOXP3* is a more definite way to confirm the diagnosis, and the presence of a normal Treg phenotype should not delay the diagnosis of IPEX syndrome [14, 15].

As of 2018, there have been around 70 mutations identified, including that of our patient [15–17].  Table 3 shows the clinical presentation of other males reported so far with the same mutation as found in our patient [17–19]. There is no correlation between the site of the mutation and the disease course or outcome, and the same genotype can present with variable phenotypes [10], and our case offers further support for this finding [20]. Overall, the lack of genotype-phenotype correlation may reflect the complex intracellular interactions of FOXP3 and strongly suggests the role of epigenetic regulation in determining the clinical picture and outcome [10, 21, 22].

If untreated, IPEX syndrome can be fatal within the first two years of life [1, 6, 14]. Hematopoietic stem cell transplant (HSCT) is an effective therapy for IPEX syndrome, despite its commonly associated complications [6, 14]. Different intensity conditioning regimens have been used with reduced intensity conditioning regimens being most successful with less morbidity and mortality [23, 24]. However, it increases the risk for graft rejection as in our patient. Mixed chimerism was detected in one-third of the patients resulting in disease remission only in the half of them, especially with full-donor Treg cell origin, such as in our patient. Early HSCT carries more favorable outcome, as some of the endocrine organs can be damaged permanently if not treated early in course of the disease [14, 25]. Early HSCT in our patient prevented every autoimmune complication; autoimmune hemolytic anemia was secondary to EBV reactivation-induced immune dysregulation or graft-versus-host disease rather than a manifestation of IPEX syndrome. There are new evolving treatments such as gene therapy which may offer a more definite cure in the future [10].

In conclusion, IPEX syndrome should be considered in young male children with severe intractable diarrhea even in the absence of eczema and other autoimmune endocrinopathies, regardless of the family history. Sequencing of the *FOXP3* gene should always be carried out to look for mutations even in the face of normal FOXP3 protein expression in the Treg cell. A timely diagnosis is essential to offer HSCT in this potentially fatal disorder.

## Figures and Tables

**Table 1:** Results of initial laboratory investigations.

	Result	Normal values
Hemoglobin	67	96–124 g/L
White blood cells	20.51	6.5–13.3 × 10^9^/L
Platelet	508	244–529 × 10^9^/L
pH	7.31	7.35–7.45
pCO_2_	41.7	35–48 mmHg
Base excess	−4.7	−4.0–2.0 mmol/L
HCO_3_	12.8	16–24 mmol/L
Total protein	43.9	62–78 g/L
Albumin	21.7	36–49 g/L
Glucose	5.4	3.3–5.6 mmol/L
IgG	3.45	0.80–5.12 g/L
IgA	0.45	0.03–0.42 g/L
IgM	0.20	0.23–0.96 g/L
IgE	95	1–110 IU/mL
Stool calprotectin	99	<50 mcg/g

**Table 2:** Lymphocyte subsets before and after the hematopoietic stem cell transplant (HSCT).

	CD19^+^ B cells	CD3^+^ T cells	CD4^+^ T cells	CD8^+^ T cells	CD4^+^25^+^127^low+^ Treg cells	CD3^+^4^+^45RA^+^ naïve T cells	CD3^−^16^+^56^+^ NK cells
At onset of disease (6 months of age)	12%, 1,430 cells/mcL (50–75pc)	82%, 9,700 cells/mcL (>95pc)	63%, 7,500 cells/mcL (>95pc)	17%, 2,000 cells/mcL (90–95pc)	8% of CD4^+^ T cells, 344 cells/mcL (10–90pc)	66%	5%, 600 cells/mcL (50–75 pc)
3 mo after the first HSCT (13 months of age), 15% donor cells on total chimerism	0%	61%, 1,150 cells/mcL (<5pc)	19%, 360 cells/mcL (<5pc)	27%, 530 cells/mcL (10–25pc)	ND	2%	29%, 560 cells/mcL (75pc)
24 mo after the second HSCT (3.5 years of age), 65% of donor cells on total chimerism	11%, 250 cells/mcL (<5pc)	81%, 1,830 cells/mcL (25pc)	47%, 1,000 cells/mcL, (25pc), 78% donor	22%, 490 cells/mcL (5–10pc), 97% donor	4% of CD4^+^ T cells, 40 cells/mcL (10–90pc), 91% donor	48%	3%, 70 cells/mcL (<5pc)

**Table 3:** Male patients reported with IPEX syndrome with a mutation in *FOXP3* gene c.1190G > A (p. R397Q).

Case	Age at onset of disease	Organ involvement			Others	Reference #
		Enteropathy	Endocrinopathy	Dermatitis		
1	4 years	+	Type 1 diabetes	+	−	18
2	1 month	+	−	+	−	19
3	6 years	+	−	−	Failure to thrive, recurrent infections (*Clostridium difficile*, *Candida albicans*, and *Mycoplasma pneumoniae*)	20
Our patient	2 months	+	−	−	Failure to thrive	
